# The Assessment of Patient-Reported Outcomes in the Medical Management of Patients With Benign Prostatic Hyperplasia

**DOI:** 10.7759/cureus.67027

**Published:** 2024-08-16

**Authors:** Malik Adil Mehmood, Shah Jehan, Issa Khan, Muhammad Ilyas, Usama Khan, Wajid Ali

**Affiliations:** 1 Urology, Institute of Kidney Diseases, Peshawar, PAK; 2 Urology, Maqsood Medical Complex (MMC) General Hospital, Peshawar, PAK; 3 Urology, Nizwa hospital, Nizwa, OMN; 4 General Practice, Category D Hospital, Razmak, PAK; 5 Surgery, Hayatabad Medical Complex, Peshawar, PAK

**Keywords:** benign prostate hyperplasia impact index, international prostate symptom score, patient-reported outcome, conservative medical management, benign prostate enlargement

## Abstract

Background

Benign prostatic hyperplasia (BPH) is a prevalent condition that a large portion of the male population develops with aging, in which the prostate gland enlarges and results in urinary symptoms.

Objective

The objective of this article is to assess patient-reported outcomes (PRO) of medical management of benign prostate hyperplasia in terms of international prostate symptoms score (IPSS), BPH impact index (BPHII), and treatment satisfaction score (TSS).

Methods

This descriptive study included 114 patients who received medical management for BPH during the period 5^th^ May 2021 till 30^th^ December 2023, at the Department of Urology, Institute of Kidney Disease Peshawar. Patient-reported outcomes were measured in terms of IPSS, BPHII, and TSS. Readings were recorded at the start of treatment and at three months of treatment and then compared. Data was analyzed using SPSS v.25 (IBM Inc., Armonk, New York).

Results

The mean age of the patients was 64.33 ± 6.12 years. The mean prostate size was 77.35 ± 12.83 ml. Overall mean pre-treatment and post-treatment IPSS was 24.82 ± 4.90 versus 15.57 ± 5.15, respectively (p-value 0.00). Mean pre-treatment and post-treatment BPHII were 11.98 ± 1.02 and 7.12 ± 2.46, respectively (p-value 0.000). The overall mean treatment satisfaction score was 6.89 ± 1.44.

Conclusion

Medical management improved symptomatology in BPH patients. This study is a step in the direction of the development of larger and longer-term PRO studies in BPH management.

## Introduction

Benign prostatic hyperplasia (BPH) is a prevalent condition that a large portion of the male population develops with aging, in which the prostate gland enlarges and results in lower urinary symptoms [1,2]. Lower urinary tract symptoms (LUTS), which can be seen in those with BPH, include frequency, urgency, nocturia, and a weak stream of urine [3]. BPH can have a substantial impact on the quality of life for patients, including effects on physical, emotional, and social domains [4,5].

The management of BPH has traditionally been based on objective clinical guides such as prostate size, urinary flow rate, and post-void residual urine volume [6]. Even so, these outcome measures may not capture the entire patient experience and rate of treatment success and failure from the patient's point of view. Recently, the significance of patient-reported outcomes (PROs) in many chronic diseases, including BPH, has been increasingly emphasized [7].

Reports are directly from patients about how they feel and function related to their health condition and its treatment [8]. These are important outcomes providing information on symptom burden, treatment efficacy, and overall quality of life, representing a broader view of the patient's health status [9]. The use of PROs in clinical practice PROs (e.g., physical, emotional, and social well-being as well as symptoms and side effects) is important to provide the full picture of a patient's health status [10]. Thus their routine integration can improve patient-centered care, communication with patients by healthcare providers, and enable personalized treatment strategies.

The current study will investigate the use of patient-reported outcomes in the medical management of BPH. Using validated PRO instruments, including the International Prostate Symptom Score (IPSS), BPH Impact Index (BPHII), and Treatment Satisfaction Score (TSS), we aim to determine how these measures might contribute to typical clinical evaluations and improve general BPH symptoms with medical treatment.

## Materials and methods

Study Design and settings

This descriptive study was carried out at the Department of Urology, Institute of Kidney Disease, Peshawar, during a period of 5^th ^May 2021 till 30^th^ December 2023.

Sampling

The study included a total of 114 male patients who fulfilled the inclusion criteria. Patients presenting with lower urinary tract symptoms (LUTS), grade 2 BPH, and prostate volume of more than 60 ml on ultrasound were included.

Exclusion criteria 

Patients with a history of prostate cancer were excluded. Patients whose radiological features were suggestive of prostate cancer, like enlargement of the gland predominantly involving the peripheral zone and having a prostate-specific antigen (PSA) of >4ng/ml, were excluded. Patients with a history of urological surgery, patients with poor cardiopulmonary status, and patients with severe comorbidities affecting the urinary tract or patient quality of life were excluded. Patients requiring surgical management were excluded.

Patients were enrolled using a non-probability consecutive sampling technique.

Outcome measurements

Outcomes were measured in terms of patient-reported outcomes (PRO) in terms of the following.

International Prostate Symptom Score (IPSS)

This tool measures seven symptoms, including frequency, urgency, nocturia, hesitancy, intermittency, weak stream, and incomplete evacuation. Each symptom is graded from 0 to 5 with a total score of 35 [11].

BPH Impact Index (BPHII)

Measures patient's quality of life. It consists of four questions with a score ranging from 0 to 13, with 0 representing no symptoms and 13 representing severe symptoms [12].

Overall Treatment Satisfaction Scale

Overall satisfaction with treatment was measured on a scale from 0 to 10, with 0 representing least or no satisfaction and 10 representing complete satisfaction.

Data collection

Patients were enrolled from the outdoor Department of Urology at the Institute of Kidney Disease Peshawar. All patients provided written informed consent. Structured interviews and self-administered questionnaires of baseline data, including demographic characteristics, clinical data, and patient-relevant outcomes, were recorded. Changes in symptoms and quality of life were recorded using IPSS, and the BPHII score was calculated. Taking into account the clinic demographics, medical management with alpha-blockers or 5-alpha-reductase inhibitors or a combination of these drugs was initiated. The choice of medication was driven by the patient's tolerability to medication and the patient's symptomatology. Patients were followed up at monthly intervals for three months. Patients were interviewed at the end of three months of treatment using a structured questionnaire comprising of questions based on IPSS and BPHII questions. The overall patient satisfaction was measured on a scale from zero to 10, as reported by the patient.

Statistical analysis

A descriptive statistical analysis was performed on data collected during the study and analyzed using Statistical Package for Social Science (SPSS) version 26.0 (IBM Inc., Armonk, New York). Descriptive statistics were used to summarize qualitative data on patient demographics and clinical characteristics, including age, BMI, residence, socioeconomic status, smoking history, comorbidities like diabetes mellitus and hypertension, and medications. Pre-treatment and post-treatment IPSS and BPH scores were compared using a paired sample t-test. Treatment satisfaction score was compared using one sample t-test, taking a value of more than five as a cut-off for better satisfaction. Linear regression model analysis was used to measure the association between predictors and patients' reported outcomes. A p-value of ≤0.05 was considered statistically significant.

## Results

Baseline clinic-demographic parameters are summarized in Table 1. The mean age of the patients was 64.33 ± 6.12 years. The majority of the patients were below 65 years of age (n=76, 66.7%). The mean BMI was 23.21 ± 1.67 kg/m2. Thirty-five patients (30.7%) were obese (BMI 24.0 kg/m2 or above). The mean prostate size was 77.35 ± 12.83 ml, and 61 patients (53.5%) patients had prostate size more than 75ml. Forty-eight participants (42.1%) had a history of smoking, 34 (29.8%) were diabetic, 47 (41.2%) were hypertensive, and 26 patients (22.8%) had a history of ischemic heart disease. Forty-four patients (38.6%) were taking 5-alpha-reductase inhibitors, 30 patients (26.3%) were advised alpha-blockers, and 40 (35.1%) were receiving a combination of alpha-blocker and 5-alpha-reductase inhibitors.

**Table 1:** Frequencies and percentages of patients according to various clinic-demographic parameters (n=114)

Parameters	Subgroups	Frequency, n (%)
Age (years), n (%)	64.33 ± 6.122 (Mean±SD)	
	65 or below	76 (66.7%)
	more than 65	38 (33.3%)
Body mass index (kg/m^2^), n (%)	23.21 ± 1.67 (Mean±SD)	
	Less than 24.0	79 (69.3%)
	24.0 or above	35 (30.7%)
Residence, n (%)	Rural	62 (54.4%)
	Urban	52 (45.6%)
Smoking, n (%)	Yes	48 (42.1%)
	No	66 (57.9%)
Socioeconomic status, n (%)	Fair	49 (43.0%)
	Poor	65 (57.0%)
Diabetes, n (%)	Yes	34 (29.8%)
	No	80 (70.2%)
Hypertension, n (%)	Yes	47 (41.2%)
	No	67 (58.8%)
Ischemic heart disease, n (%)	Yes	26 (22.8%)
	No	88 (77.2%)
Complaints duration (months), n (%)	3.80 ± 1.896 (mean±SD)	
	5 or below	89 (78.1%)
	more than 5	25 (21.9%)
Prostate size (ml), n (%)	77.35 ± 12.83 (Mean±SD)	
	75 or below	53 (46.5%)
	More than 75	61 (53.5%)
Medications pattern, n (%)	Alpha blockers	30 (26.3%)
	5-alpha-reductase inhibitor	44 (38.6%)
	Combination	40 (35.1%)

The patient-reported outcomes are summarized in Table 2. The overall mean pre-treatment and post-treatment IPSS was 24.82 ± 4.90 versus 15.57 ± 5.15, respectively. The mean difference was 9.25 ± 4.77. The paired sample student t-test p-value for the mean difference was 0.000. Mean pre-treatment and post-treatment BPHII was 11.98 ± 1.02 and 7.12 ± 2.46, respectively. The mean difference was 4.86 ± 2.64 (p-value=0.000). The overall mean treatment satisfaction score was 6.89 ± 1.44. The p-value for one sample t-test value, taking a value of 5 as a cut-off for comparison, was 0.000, i.e., <0.05, hence statistically significant.

**Table 2:** Mean and standard deviation of patients according to patient reported-outcomes (n=114) IPSS - International Prostate Symptom Score (range: 0-35); BPHII - Benign Prostatic Hyperplasia Impact Index (range :0-13); TSS - Treatment Satisfaction Scale (range: 0-10) Paired sample t-test and one sample t-test were applied to the above data

	Mean	Standard deviation	Mean difference (pre-post)	Standard deviation for mean difference	p-value
Pre-treatment IPSS	24.82	4.903	9.254	4.774	0.000
Post-treatment IPSS	15.57	5.156			
Pre-treatment BPHII	11.98	1.023	4.861	2.648	0.000
Post-treatment BPHII	7.12	2.468			
TSS	6.89	1.447			0.000

Linear regression model analysis was used to control the effect modifiers, as shown in Table 3, Table 4, and Table 5. None of the factors were found to be significantly associated with the BPHII score. Medication patterns and BMI were found to be significantly associated with the IPSS score. The p-value for medication pattern was 0.035 and 0.000 for BMI. However, no factors were found to significantly affect treatment satisfaction scores.

**Table 3:** Linear regression model analysis for the association between predictors and outcomes for BPHII (n=114) BPHII - Benign Prostate Hyperplasia Impact Index Score Linear regression model analysis was used for the above data.

Predictors	Standardized coefficients	t	p-value	95.0% confidence interval	
	Beta			Lower bound	Upper bound
(Constant)		1.962	.052	-.066	12.450
Age (years)	-.030	-.305	.761	-1.280	.940
Residence	-.113	-1.005	.317	-1.782	.583
Smoking	-.062	-.573	.568	-1.486	.820
Socioeconomic status	.076	.711	.479	-.725	1.535
Diabetes	.075	.688	.493	-.818	1.687
Hypertension	-.077	-.663	.509	-1.639	.818
Ischemic heart disease	-.010	-.097	.923	-1.359	1.232
Medication pattern	-.052	-.437	.663	-.973	.622
Complaints duration (months)	-.080	-.768	.444	-1.831	.809
Body mass index (kg/m^2^)	-.017	-.166	.869	-1.222	1.034
Prostate size (ml)	.098	.991	.324	-.521	1.560

**Table 4:** Linear regression model analysis for the association between predictors and outcomes for IPSS (n=114) IPSS - International Prostate Symptoms Score Linear regression model analysis was used for the above data.

Predictors	Standardized coefficients	t	p-value	95.0% confidence interval	
	Beta			Lower bound	Upper bound
(Constant)		.324	.746	-8.464	11.774
Age (years)	-.060	-.673	.503	-2.403	1.186
Residence	.016	.162	.872	-1.756	2.069
Smoking	.057	.582	.562	-1.318	2.411
Socioeconomic status	-.074	-.771	.442	-2.538	1.117
Diabetes	.054	.545	.587	-1.469	2.583
Hypertension	-.120	-1.154	.251	-3.142	.831
Ischemic heart disease	.047	.502	.617	-1.565	2.625
Medication pattern	.227	2.132	.035	.097	2.674
Complaints duration (months)	.060	.639	.524	-1.447	2.821
Body mass index (kg/m^2^)	.405	4.536	.000	2.347	5.994
Prostate size (ml)	-.055	-.613	.541	-2.203	1.162

**Table 5:** Linear regression model analysis for the association between predictors and outcomes for TSS (n=114) TSS - Treatment Satisfaction Score Linear regression model analysis was used for the above data.

Predictors	Standardized coefficients	T	p-value	95.0% confidence interval	
	Beta			Lower bound	Upper bound
(Constant)		4.287	.000	3.933	10.707
Age (years)	.122	1.236	.219	-.227	.975
Residence	.000	.000	1.000	-.640	.640
Smoking	-.084	-.780	.437	-.870	.379
Socioeconomic status	-.101	-.956	.341	-.907	.317
Diabetes	.002	.020	.984	-.671	.685
Hypertension	.016	.143	.886	-.617	.713
Ischemic heart disease	-.078	-.761	.449	-.970	.432
Medication pattern	-.050	-.423	.674	-.523	.340
Complaints duration (months)	-.077	-.746	.457	-.983	.446
Body mass index (kg/m^2^)	.041	.421	.675	-.481	.740
Prostate size (ml)	.150	1.527	.130	-.130	.997

## Discussion

Patient-reported outcomes (PRO) subjectively measure the patient's overall health status with no control from the physician. This study measured patient-reported outcomes with medical management in BPH. The mean pre-treatment international prostate symptoms score (IPSS) was 24.82 ± 4.90, and post-treatment (at three months) the score was 15.57 ± 5.15. The mean baseline IPSS score in a study by Schumacher et al. was 19.0 ±7.2, while post-treatment, the score reduced to 6.2 ± 5.7 [13]. Though a reduction in score was observed in both studies, the latter reported a much lower score. This may be attributed to lower baseline IPSS in the latter. Moreover, the treatment modality in the later study was Holmium Laser Enucleation of the Prostate (HoLEP), which has a much better treatment response than the medical management employed in our study. It is vital to mention that the follow-up period in the later study was much longer (18 months) compared to three months in our study. In another study by Mandal et al., the mean IPSS score reduced from baseline 18.86 ± 5.53 to 11.76 ± 3.94 after six months of treatment (p<0.001), similar to our study [14].

The mean baseline BPH impact index in our study was 11.98 ± 1.02 and 7.12 ± 2.46, respectively. The mean difference was 4.86 ± 2.64 (p-value=0.000). These observations were similar to the results of the study carried out by Mandal et al. [12]. The mean BPH impact index in the later study was 9.65 ± 2.59, which was slightly lower than our study. The mean BPHII at six-month follow-up was 5.89 ± 2.24. The mean difference in baseline and follow-up BPHII was similar to our findings (p-value<0.001). Roehrborn and colleagues showed that improvement in scores can be further improved with more prolonged follow-up [15].

The study highlighted various clinic-demographic parameters of the patients. The mean age of the patients in this study was 64.33 ± 6.122 years, which is consistent with the typical age-related to BPH studies. For instance, Ikemoto et al. measured a slightly lower mean age of around 65 years in their population-based study of BPH prevalence and symptoms [16]. Unsurprisingly, the prevalence of BPH becomes more common with age, and hence, in our study, the distribution of age group follows a typical pattern, observed widely across studies published in literature where most patients were aged between 60-79 years as documented by Gacci et al., which stated as the proportion of men over age 60 increases prostatism compliance is on the rise as aftermath [17].

The mean BMI in our study was 23.21 ± 1.67 kg/m2. Thirty-five patients (30.7%) were obese (BMI 24.0 kg/m2 or above). Our cohort had a BMI distribution that was similar to larger epidemiological studies. In the study by Parsons et al., a significant percentage of BPH patients with overweight and obesity was also reported, potentially confirming the association between BMI and BPH symptom severity [18].

In this study, sociodemographic distribution was similar to the demographic spreads reported in studies, among which educational attainment might influence health-seeking behavior and disease management, as postulated by Vuichoud and Loughlin [19]. Egan reported a higher proportion of the urban population, as opposed to our study, indicating that rural populations are often harder to reach when it comes to clinical research [20]. This difference may be attributed to the rapid migration of the population from rural to urban areas. The distribution of patients according to socioeconomic status was similar to that reported by Parsons et al., emphasizing the influence of socioeconomic factors on health outcomes [18].

The distribution of medication use is closely similar to previous studies. For instance, Roehrborn et al. identified similar medication use patterns where alpha-blockers were the most frequently prescribed medications, but this is not always the case [15].

This study is not without limitations. Firstly, it was an observational study with no control group. An analytical study design with a control arm could have improved the significance of the results. Secondly, the study was carried out at a single center, which limits the generalizability of the study. Thirdly, patient-reported outcomes are solely dependent on patient recall. The patient's mental health could potentially interfere with the results. Fourthly, compliance to medications in terms of dose and frequency wasn't measured using a standardized medication adherence measuring tool. Finally, the scope of the results could have been expanded by prolonging the follow-up period.

## Conclusions

The study provides an overview of the clinic demographics parameters of patients with benign prostate hyperplasia. The study also provides insights into the outcomes of medical management of BPH in terms of patient-reported outcomes, including IPSS, BPHII, and TSS scores. Significant improvement in IPSS and BPHII was observed after three months of medications (alpha-adrenergic blockers, 5-alpha-reductase inhibitors, or combination therapy) use for BPH. Patients showed their satisfaction with treatment, as evident by a significant Treatment Satisfaction Score.
